# Monocyte Maturation Mediators Upregulate CD83, ICAM-1 and MHC Class 1 Expression on Ewing’s Sarcoma, Enhancing T Cell Cytotoxicity

**DOI:** 10.3390/cells10113070

**Published:** 2021-11-08

**Authors:** Emilie Biele, Sebastian J. Schober, Carolin Prexler, Melanie Thiede, Kristina von Heyking, Hendrik Gassmann, Jennifer Eck, Busheng Xue, Stefan Burdach, Uwe Thiel

**Affiliations:** 1 Department of Pediatrics, Children’s Cancer Research Center, Kinderklinik München Schwabing, School of Medicine, Technical University of Munich, 80804 Munich, Germany; s.schober@tum.de (S.J.S.); carolin.prexler@tum.de (C.P.); melanie.thiede@tum.de (M.T.); kristina.heyking@tum.de (K.v.H.); hendrik.gassmann@tum.de (H.G.); jennifer.eck@tum.de (J.E.); busheng.xue@tum.de (B.X.); stefan.burdach@tum.de (S.B.); 2 German Cancer Consortium (DKTK), German Research Center (DKFZ), Partner Site Munich, 80336 Munich, Germany

**Keywords:** Ewing sarcoma, CD83, immunotherapy, CHM1^319^-specific TCR-transgenic T cells

## Abstract

Ewing’s sarcoma (EwS) is a pediatric solid tumor entity with low somatic mutational burden and a low rate of tumor-infiltrating T cells, indicating a low extent of immunogenicity. In EwS, immunogenicity may furthermore be significantly diminished by a predominantly M2 macrophage driven pro-tumorigenic tumor microenvironment. In the past, we demonstrated that CHM1^319^-specific TCR-transgenic T cells are able to control EwS growth in a preclinical mouse model as well as in a patient with metastatic disease. However, new adjuvant techniques to induce long lasting and curative CHM1^319^-specific TCR-transgenic T cell-mediated anti-tumor responses are needed. In this work, we sought to identify a technique to improve the cytotoxic effect of CHM1^319^-specific TCR-transgenic T cell by altering the immunogenic cell surface marker expression on EwS cell lines using different cytokines. We demonstrate that TNF, IL-6, IL-1β and PGE_2_ cause pro-immunogenic CD83, MHC class I and II as well as ICAM-1 upregulation in EwS cell lines. This observation was associated with significantly improved recognition and killing of the tumor cells by EwS-specific CHM1^319^/HLA-A*02:01-restricted TCR-transgenic T cells. Conclusively, we demonstrate that the induction of an inflammatory signature renders EwS more susceptible to adoptive T cell therapy. TNF, which is upregulated during inflammatory processes, is of particular translational interest as its secretion may be induced in the patients e.g., by irradiation and hyperthermia in the clinical setting. In future clinical protocols, this finding may be important to identify appropriate conditioning regimens as well as point of time for adoptive T cell-based immunotherapy in EwS patients.

## 1. Introduction

Ewing’s sarcoma (EwS) represents a highly malignant pediatric solid tumor entity. EwS carries a low somatic mutational burden and a low rate of tumor-infiltrating T cells, implying a low immunogenicity. Upregulation of the endochondral bone protein Chondromodulin-I (CHM1) in EwS is driven by the fusion oncogene EWS-FLI1. In this context, CHM1 has been identified as a promising target antigen for immunotherapy in EwS patients [1,2,3,4]. Our research group has previously succeeded in generating CHM1^319^-specific TCR-transgenic T cells that showed anti-tumoral effects preclinically in vitro and in vivo [5]. In the past, we demonstrated clinical efficacy of CHM1^319^-specific TCR-transgenic T cells in at least one out of three treatment-refractory EwS patients, without causing side effects despite high numbers of adoptively transferred T cells. In at least one out of three patients, CHM1^319^-specific TCR-transgenic T cell homing to affected bone marrow could be monitored. Tumor regression was observed without causing symptoms of graft versus host disease (GvHD) [6].

CD83 is a transmembrane glycoprotein of the immunoglobulin superfamily and is predominantly expressed on several activated immune cells [7]. Two isoforms have been identified: soluble CD83 (sCD83) and membrane-bound CD83 (mCD83). While the latter is primarily known to be a highly specific surface marker on mature dendritic cells (DCs), sCD83 is attributed with mainly immunosuppressive properties [8,9]. HSV-1-based studies have shown the role of CD83 as a T cell enhancer by demonstrating a correlation between decreased CD83 surface expression on HSV-1 infected DCs with an impaired CD8^+^ T cell stimulation [10]. Furthermore, more recent studies have demonstrated an expression of a CD83 ligand expression on CD8^+^ T cells upon TCR activation which, when binding to CD83 on APC, results in increased antigen-specific CD8^+^ T cell numbers [11].

In this analysis, we hypothesized that an immunogenic signature can be induced using different cell mediators in EwS cell lines. In particular, we hypothesized an immunogenic effect of cell mediators used in the differentiation of dendritic cells out of CD14+ myeloid cells. We hypothesized that mCD83 up-regulation in EwS is feasible, causing significantly improved susceptibility of HLA-A*02:01 positive EwS cell lines when treated with CHM1^319^-specific TCR-transgenic T cells in vitro.

## 2. Materials and Methods

### 2.1. Cell Lines and Culturing

**Figure 1:**
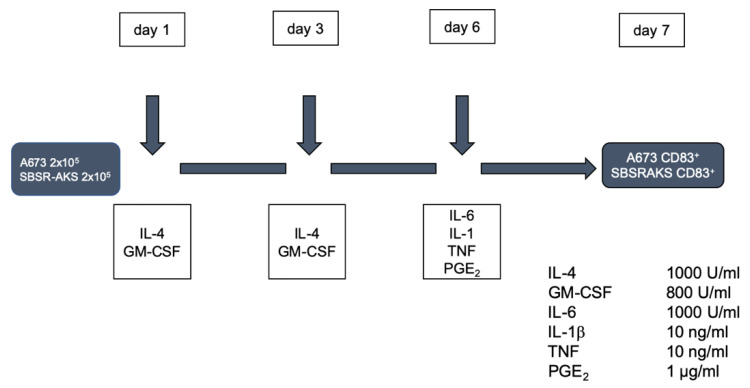
Protocol for culturing EwS cell lines A673 and SBSR-AKS in vitro using IL-4, GM-CSF, IL-6, IL-1β, TNF, and PGE.

HLA-A*02:01^+^ EwS line A673 and HLA-A*02:01^-^ EwS cell line SBSR-AKS were cultured in medium containing X-Vivo 15 and 1% human AB serum. The protocol applied to the ‘supplemented’ EwS cell lines is generally used to differentiate CD14^+^ monocytes into DCs [2]. On day one, medium with 2 × 10^5^ tumor cells was supplemented with 1000 U/mL IL-4 (R&D Systems, Minneapolis, MN, USA) and 800 U/mL GM-CSF (Sanofi, Paris, France). Medium was renewed at the same concentrations on day three. On day six, in accordance to DC maturation protocols, the medium was again renewed and supplemented with IL-6 (1000 U/mL), IL-1β (10 ng/mL), TNF (10 ng/mL) and PGE_2_ (1 µg/mL) (all R&D Systems). Further analyses commenced at day 7 (Figure 1). Controls were cultured in RPMI 1640 medium supplemented with 10% fetal calf serum (FCS), 100 U/mL penicillin, 100 mg/mL streptomycin (Gibco, Thermo Fisher Scientific, Waltham, MA, USA) without addition of cytokines (termed ‘control’).

### 2.2. Fluorescence-Activated Cell Sorting (FACS)

Surface expression of CD83 (APC-Vio770), MHC class I and II (APC), ICAM-1 (PE), PD-L1 (PE) and PD-L2 (APC-Vio770) were determined by flow cytometry analysis using MACSQuant^®^ Analyzer 10 (Miltenyi Biotec, Bergisch-Gladbach, Germany). The antibodies ICAM-1, PD-L1, PD-L2, CD83, MHC class I and II and respective isotypes were purchased from Miltenyi Biotec. Respective APC-, APC-Vio770- or PE-conjugated IgG isotypes served as staining controls. Control EwS cell lines cultivated in RPMI 1640 medium (10% FCS, P/S) constituted respective control groups.

We hypothesized that only the cell mediators responsible for differentiation may cause an immunogenic effect on EwS cell lines. To identify driver cytokines of CD83 induction, FACS analyses were performed on A673 EwS cells cultivated in medium containing (1) either only one of the DC maturation cytokines (IL-6, IL-1β, TNF or PGE_2_), respectively, or (2) three out of four DC maturation cytokines, respectively. The reason why we chose this approach was to juxtapose the possible immunogenic effect in respective contrast to the effect of the three remaining cell mediators in order to screen for the strongest impact of each single mediator. To determine the point of time of CD83 surface upregulation, we performed FACS analyses at day four, day six, and day seven of the maturation protocol (Figure 1). Prior to sample acquisition, cells were stained using propidium iodide solution to distinguish dead and alive cells.

FACS data was analyzed using FlowJo™ 10.7.2 Software (BD Biosciences, San Jose, CA, USA). For analysis of the flow cytometry data, gates were set to identify living cells as well as single cells only. Furthermore, quadrants were set according to the respective APC-, APC-Vio770- and PE isotype-labelled control samples.

### 2.3. Retroviral T Cell Transduction, Purification, Expansion and Culture Methods

The CHM1^319^/HLA-A*02:01-specific TCR transgene was introduced into T cells using the retroviral vector pMP-71 [12,13]. Purified CD8^+^ T cells were activated with 3 µg/mL OKT3 according to manufacturer’s recommendation and 100 U/mL IL-2 (Novartis) was added for T cell proliferation. Two days later, spin infection with virus supernatant from the packaging cell line RD114 was performed. On the next day, T cells were re-infected with the same approach. After verifying successful transduction via FACS staining, T cell subsets were purified with anti-phycoerythrin (PE) magnetic beads (Miltenyi Biotec) coupled to CHM1^319^/HLA-A*02:01-specific PE labeled multimers as previously published [6]. Upon generation, T cells were frozen and stored in liquid nitrogen for further use.

### 2.4. CHM1^319^-Specific TCR-Transgenic T Cell-Mediated EwS Recognition and Lilling

CHM1^319^/HLA-A*02:01-specific recognition and killing of A673 EwS cells cultured in cytokine supplemented medium compared to the control group cultured in standard control medium, was assessed using interferon-γ and granzyme B ELISpot assays as previously described [2]. CHM1^319^-specific TCR-transgenic T cells were thawed and expanded by adding 100 U/mL rhIL-2 and 2 ng/mL rhIL-15. T2 cells primed with influenza and CHM1 peptides served as negative and positive control groups, respectively. For the interferon-γ ELISpot assay 1000 and 5000 CHM1^319^/HLA-A*02:01-specific T cells and 20,000 target cells were used. For granzyme B ELISpot assays, effector to target ratios were titrated from 2.5:1 to 0.156:1. All experiments were performed in sextuplicates for interferon-γ assays and triplicates for granzyme B assays according to the manufacturer’s protocols, respectively.

### 2.5. RNA Gene Expression Analysis

RNA from A673 and SBSR-AKS cells cultivated in supplemented–versus control medium was isolated using the extraction reagent TRIzol and the Zymo Research Direct-zol™ RNA MiniPrep Kit. The extracted RNA served as a template for cDNA synthesis (according to the Ambion^®^ WT Expression Kit) which was fragmented and labelled following the Affymetrix^®^ GeneChip^®^ WT Terminal Labelling and Hybridization protocol prior to its injection onto a GeneChip^®^ Probe Array. Alterations in gene expression were compared and analyzed by gene set enrichment analysis and visualized using iSEE (Bioconductor). Affymetrix-based RNA expression analyses were performed in order to evaluate RNA signatures induced by DC medium conditioning in A673 and SBSR-AKS EwS cell lines. Gene expression analyses were illustrated using iSEE [13].

### 2.6. Statistical Analysis

GraphPad Prism (GraphPad Software, San Diego, CA, USA) was used to calculate mean and standard deviation of the mean (SEM). Differences between two groups were determined using the student’s *t*-test with *p*-values < 0.05 being considered statistically significant.

## 3. Results

### 3.1. Supplemented A673 and SBSR-AKS EwS RNA Expression Signatures Indicate Upregulation of Immune Response Drivers CD83, ICAM-1, and MHC Class I

Heatmap analyses showed an increase of CD83 and ICAM-1 expression in both DC medium supplemented versus control A673 and SBSR-AKS cell lines (Supplemental Figure S1A). SBSR-AKS cells showed a distinct increase in both MHC class I and MHC class II expression, whereas A673 demonstrated up-regulation of MHC class I only. In both cell lines supplemented with DC medium, a considerable upregulation of ICAM-1 could be observed. Although the changes displayed on RNA level are subtle, they are in line with the findings obtained by flow cytometry (Supplemental Figure S1B).

### 3.2. mCD83 Is Increased after Co-Cultivation with DC Maturation Cytokines in Flow Cytometry

**Figure 2:**
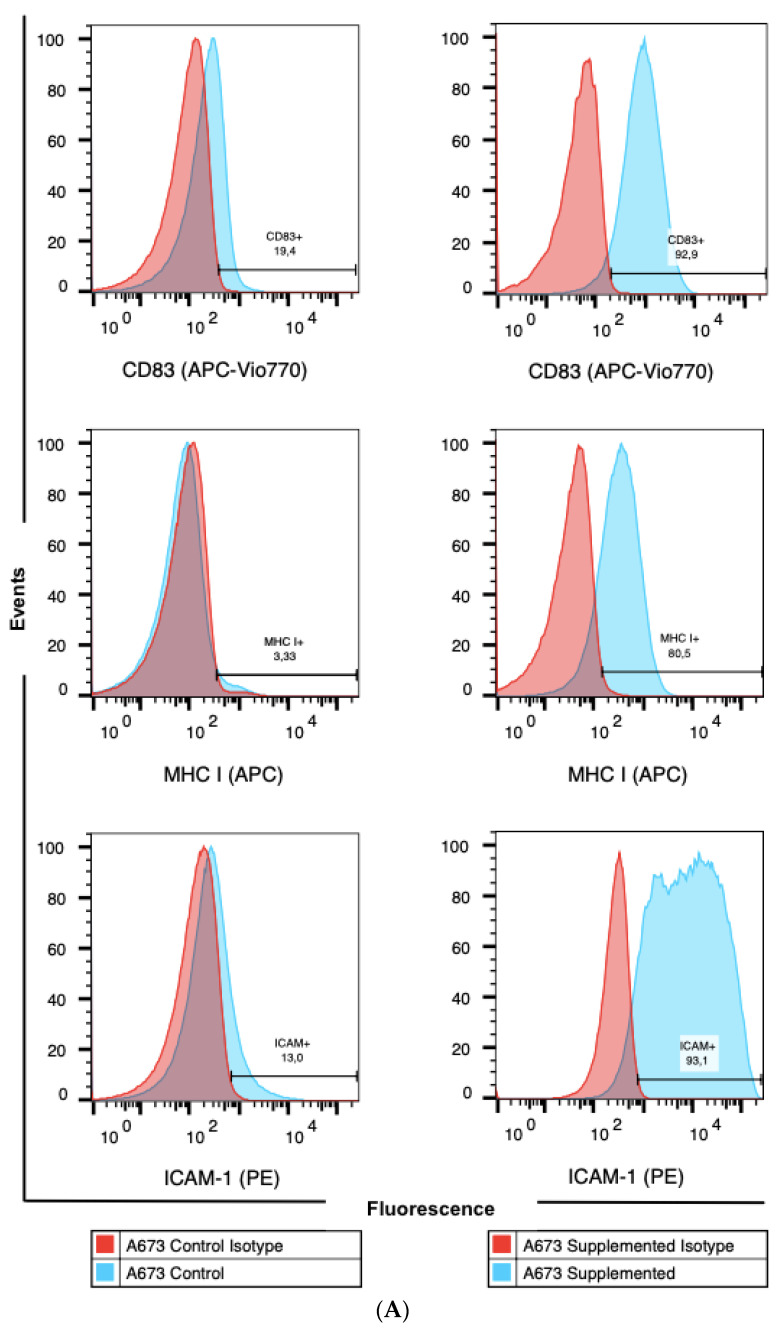

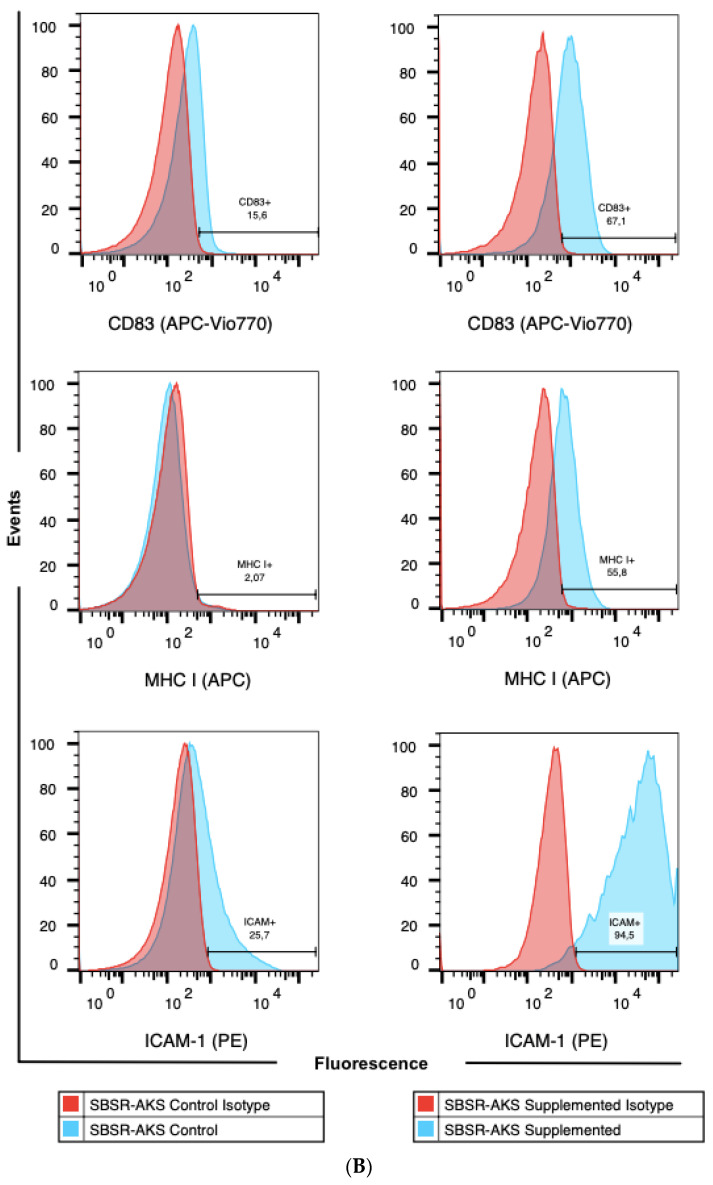
Upregulation of immunogenic cell surface markers by DC cytokines. (**A**) A673 CD83, MHC class I, and ICAM-1 expression are upregulated in supplemented (right) versus control cells (left). The gates were set according to isotype-labeled control samples. (**B**) SBSR-AKS CD83, MHC class I and ICAM-1 expression are upregulated in supplemented (right) versus control cells (left). Gates were set according to the IgG-isotype control (red histogram) and compared to the antibody-labelled cells (blue histogram).

On day seven of cell mediator treatment (Figure 1), we assessed the cell surface profiles of A673 and SBSR-AKS cultivated in supplemented medium compared to control medium using flow cytometry analysis. Figure 2 demonstrates CD83 expression of both cytokine supplemented cell lines A673 and SBSR-AKS compared to controls. An increase of the DC maturation markers CD80 could not be seen in either cell line. Both cytokine supplemented A673 and SBSR-AKS cell lines showed an increase of MHC class I compared to the control group, although the up-regulation noted on A673 was more distinct. There was no change in MHC class II expression (Supplemental Figure S3). Both A673 and SBSR-AKS showed an increase in ICAM-1 surface expression in supplemented cells compared to controls. Surface PD-L1 and PD-L2 only showed a minor increase in supplemented A673 cells compared to controls. There was no change in SBSR-AKS (Supplemental Figure S3).

### 3.3. TNF Is a Potent Driver of CD83 Induction on EwS Cell Lines

**Figure 3:**
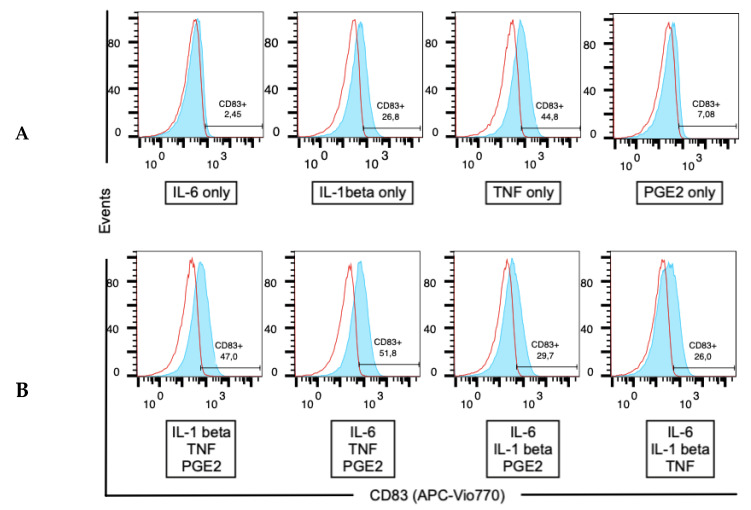
CD83 Flow cytometry (**A**) CD83 upregulation on A673 cells after addition of either IL-6, IL-1β, TNF and PGE_2_ only, respectively after two doses of IL-4 and GM-CSF (day six of in vitro culture). (**B**) CD83 upregulation on A673 cells after addition of three out of four cytokines (IL-6, IL-1β, TNF and PGE_2_) of the DC maturation cocktail on day six. Quadrants were set after staining a control sample for APC-Vio770 isotypes (red open histogram) for each cytokine group tested (blue histogram). All experiments were performed in independent replicates.

A673 cells treated with TNF alone showed the most prominent CD83 upregulation compared to IL-6, PGE_2_ and IL-1β treatment alone, respectively. Correspondingly, FACS analysis of A673 treated with three out of four cytokines showed a varying increase in CD83 surface expression in all groups (Figure 3). This experiment was conducted three times. The *p*-values obtained showed a difference in CD83 upregulation between the use of a single cytokine and the control group: the difference between the use of IL-6 only, IL-1β only and PGE_2_ only compared to the use of control medium were *p* = 0.36, 0.12, and 0.51, respectively and hence rendered as not significant. When comparing the use of TNF only with the control medium, we obtained a *p*-value of 0.01, yielding a statistically significant difference between both groups. There was no difference in CD83 upregulation between standard TNF dose versus fivefold TNF dose (Supplemental Figure S2).

### 3.4. Increased CD83 Surface Expression Is Associated with Enhanced T Cell-Mediated Specific EwS Recognition

**Figure 4:**
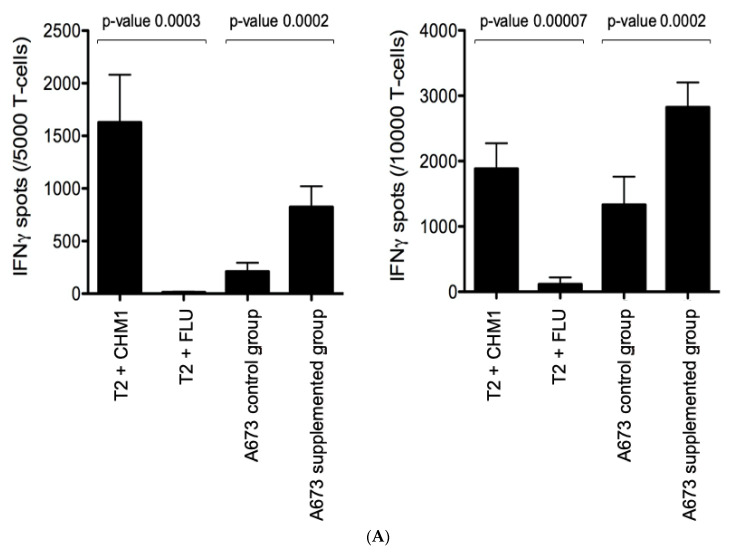

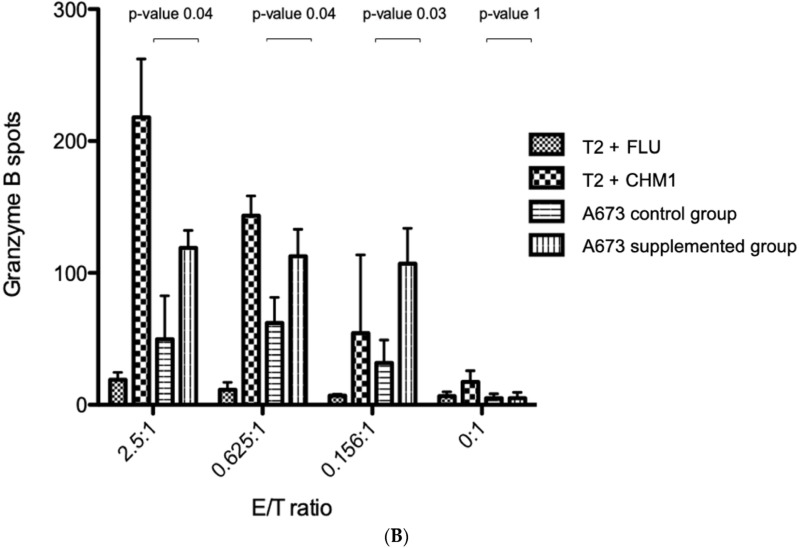
CHM1^319^/HLA-A*02:01-specific T cell-mediated recognition and killing of A673 EwS cell lines and T2 pulsed cells in interferon-γ and granzyme B ELISpot assays. Error bars indicate standard deviation of sextuplicates. (**A**) Interferon-*γ* ELISpot assays; A673 and CHM1^319^ peptide-loaded T2 cells are specifically recognized by CHM1^319^/HLA-A*02:01-specific T cells as compared to T2 cells loaded with influenza-derived control peptides. A673 supplemented with DC maturation cytokines, induce a significantly higher interferon-γ secretion as compared to control A673 cells when 5.000 or 10.000 effector cells were used, respectively (both *p* < 0.05). (**B**) Granzyme B ELISpot assay; Dose-dependent CHM1^319^/HLA-A*02:01-specific T cell-mediated killing of A673 and T2 cells loaded with CHM1^319^ compared to influenza control peptide loaded T2 cells (FLU). A673 supplemented with DC generation-associated cell mediators induce a significantly higher granzyme B secretion compared to control A673 cells in effector to target ratios of 2.5 (*p* = 0.04), 0.625 (*p* = 0.04) and 0.156 (*p* = 0.03).

We evaluated the effect of CD83 upregulation on the cytotoxic potential of CHM1^319^/HLA-A*02:01-specific T cells [6] directed against EwS cell lines. A673 tumor cells were cultivated in supplemented medium for six days according to the previously mentioned DC maturation protocol (Figure 1). On day seven, the interferon-γ and granzyme B ELISpot assays were performed. In interferon-γ assays, A673 as well as T2 cells loaded with CHM1^319^ antigen were specifically recognized by CHM1^319^/HLA-A*02:01-specific T cells as compared to T2 cells loaded with influenza-derived control peptide. A673 supplemented with DC maturation cytokines, however, induced a significantly higher interferon-γ secretion compared to control A673 cells, indicative of a stronger immune response upon co-cultivation. *P*-values were *p* < 0.05 when 5000 and 10,000 effector cells were used (Figure 4A). The granzyme B assays demonstrate dose-dependent CHM1^319^/HLA-A*02:01-specific T cell-mediated killing of T2 cells loaded with CHM1^319^ compared to T2 cells loaded with influenza-derived control peptide. A673 killing was equally dose-dependent [14]. A673 supplemented with DC maturation cytokines, however, induced a significantly higher granzyme B secretion indicative of tumor cell killing compared to control A673 cells in effector to target ratios of 2.5 (*p* = 0.04), 0.625 (*p* = 0.04) and 0.156 (*p* = 0.03), confirming stronger anti-tumor effects when A673 was supplemented (Figure 4B). These findings are in line with the observed elevation of CD83 and MHC class I expression stated previously (Figure 2).

## 4. Discussion

According to a recent analysis performed by Gröbner et al., pediatric cancers can be genetically subdivided into two classes: entities with low mutation rates and entities with structural/copy-number variants [15]. In the first group, lack of somatic mutations may result in low immunogenicity impairing immunotherapeutic approaches such as adoptive T cell treatment and/or immune-checkpoint inhibition [15,16]. However, in order to implement successful immunotherapy in patients with pediatric tumors of this group, such as in EwS, the induction of an inflammatory microenvironment may render immunologically cold tumors susceptible to T cell-mediated immunotherapy.

In the present study, we sought to identify cytokines with the potential to render EwS more susceptible to treatment with CHM1^319^/HLA-A*02:01-specific T cells. Therefore, we treated EwS cell lines with different cytokines routinely used for DC generation of CD14^+^ monocytes. Interestingly, we observed a wide spectrum of upregulated immunogenic genes and surface markers upon treatment associated with immunogenicity towards T cells, such as CD83, ICAM-1 and MHC class I upregulation both on RNA level and protein level. Given these observations, we sought to identify the cytokine responsible for inducing such immunogenicity in EwS.

ICAM-1, also known as CD54, is a transmembrane glycoprotein of the Ig superfamily and is most prominently known for its co-stimulatory function enhancing the interaction between DCs and T cells [17]. ICAM-1 is commonly expressed on immune cells although expression on tumor cell surface has been demonstrated. Observations made by Bailey et al. indicate that ICAM-1 promotes T cell activation and T cell/tumor cell interaction by highly specific binding of ICAM-1 expressed on tumor cells and the corresponding lymphocyte function-associated antigen-1 (LFA-1) expressed on lymphocytes [18,19].

As indicated previously, several types of cancer downregulate their cell surface expression of MHC class I over time as an immune escape mechanism by which they dampen the immune response directed against tumor tissue [20]. Studies conducted by Berghuis et al. investigating the MHC expression on EwS cell lines obtained heterogeneous results. However, they found that the majority of EwS cell lines demonstrated a substantial downregulation of MHC expression, including in metastatic tissues implying the negative effect it might bear on immunotherapy [21,22]. Hence, the upregulated MHC expression observed in our results may constitute a therapeutically exploitable way to inverse this immune escape mechanism.

Membrane-bound CD83 is a surface protein suggested to be a key immunological player in the regulation of CD4^+^ and CD8^+^ T cell responses. In immature DCs, CD83 is preformed intracellularly and is rapidly expressed on cell surface upon activation and maturation [23,24]. Furthermore, CD83 stabilizes the expression of MHC class II and CD86 on DCs [24]. Although expression of CD83 on immune cells has been described extensively, to the best of our knowledge CD83 expression on tumor cells and its role in the induction of anti-tumor responses in this context has not been elucidated in the literature. Although the exact mechanism remains unclear, we have observed an increase in CD83 expression upon treatment with cell mediators, concordant with enhanced T cell recognition and killing of EwS cell lines. However, we were able to induce an immunogenic signature using a series of mediators and pose the upregulation of CD83 as a possible mechanism of action.

Among the implemented cytokines of the DC-supplemented EwS culture media in this study, TNF was associated with most prominent CD83 upregulation. These findings are in line with previous reports identifying TNF as an activating agent for increased CD83 expression on monocyte-derived DCs and therefore a potent driver for DC maturation [25,26]. Moreover, CD83 upregulation was correlated with an improved recognition and killing of EwS cells by CHM1^319^/HLA-A*02:01-specific T cells in ELISpot assays.

In the past, studies conducted with TNF as an anti-tumor agent have yielded heterogeneous results. As a systemic agent, simultaneous application of TNF and other anti-cancer agents resulted in an increased uptake and enhanced anti-tumor efficacy. However, TNF has also shown to induce severe toxicity at doses even lower than the predicted efficient dose [27,28,29]. Hyperthermia and irradiation may cause tissue injury, tumor destruction and the secretion of proinflammatory cytokines [30,31,32]. As a result, antigen presentation is augmented via mechanisms such as antigen cross-presentation, HLA up-regulation, activation of the immune system (e.g., via IL-2, TNF and IFNγ) and expression of co-stimulatory molecules (e.g., CD40/CD40L or B7/CD28) in both tumor and in the tumor microenvironment. This immunogenic reaction upon tissue damage may lead to enhanced anti-tumor responses after adoptive transfer of tumor antigen-specific T cells even in immunologically cold tumors, such as EwS [30,33]. Interestingly, EwS itself secretes TNF upon irradiation and local hyperthermia [34,35]. Various research groups have observed an upregulation of TNF mRNA and protein levels in response to irradiation in a time-dependent manner in TNF producing EwS cell lines [29,34,36]. Consequently, the incorporation of DC maturation mediators in pre-adoptive T cell conditioning regimens in patients with advanced EwS eligible for treatment might render EwS more susceptible to immunotherapy e.g., with CHM1^319^-specific TCR-transgenic T cells.

The present results imply that it is possible to induce tumor immunogenicity in primarily non-immunogenic EwS via exogenous stimuli. Neo-adjuvant sensitizing of therapy-refractory EwS induced by triggering a local inflammation in the tumor area, for example by local irradiation or by delivery of inflammatory mediators, may be a further step towards the improvement of successful T cell-based immunotherapy. Rübe et al. showed that increased biologically active TNF levels can be induced in a dose-dependent manner in a majority of EwS cell lines by local irradiation in both in vitro and in xenograft mouse models [36]. Our observation of induction of immunogenicity in EwS cell lines may thus be of crucial importance in the planning of immunogenic conditioning regimen of patients with refractory EwS prior to adoptive T cell transfer with tumor-associated antigen (TAA)-specific TCR transgenic T cells. Hence, the translation of this observation into a clinical setting will contribute to the identification of necessary immunotherapeutic conditioning regimes, as well as an improved timing of the adoptive T cell transfer after appropriate immunogenic conditioning in future clinical trials.

## 5. Conclusions

In the present analysis, we demonstrate that EwS can be altered to become more susceptible to the effect of adoptive T cell therapy using proinflammatory cytokines conventionally used for the generation of DCs. In the future, the induction of immunogenicity prior to adoptive transfer of CHM1^319^/HLA-A*02:01-specific T cells may significantly enhance anti-EwS responses.

## Supplementary Materials

The following are available online at https://www.mdpi.com/article/10.3390/cells10113070/s1, Figure S1: RNA expression, Figure S2: CD83 flow cytometry, Figure S3: PD-L1, PD-L2 and MHC class II flow cytometry.
