# Supplementary material for: Monocyte Maturation Mediators Upregulate CD83, ICAM-1 and MHC Class 1 Expression on Ewing’s Sarcoma, Enhancing T Cell Cytotoxicity
Source: Cells. 2021 Nov 8;10(11):3070. doi: 10.3390/cells10113070 (PMC8624504; doi:10.3390/cells10113070)
Supplement: Supplementary file 1 [file cells-10-03070-s001.zip › cells-1424767-supplementary.pdf]

Supplementary Material

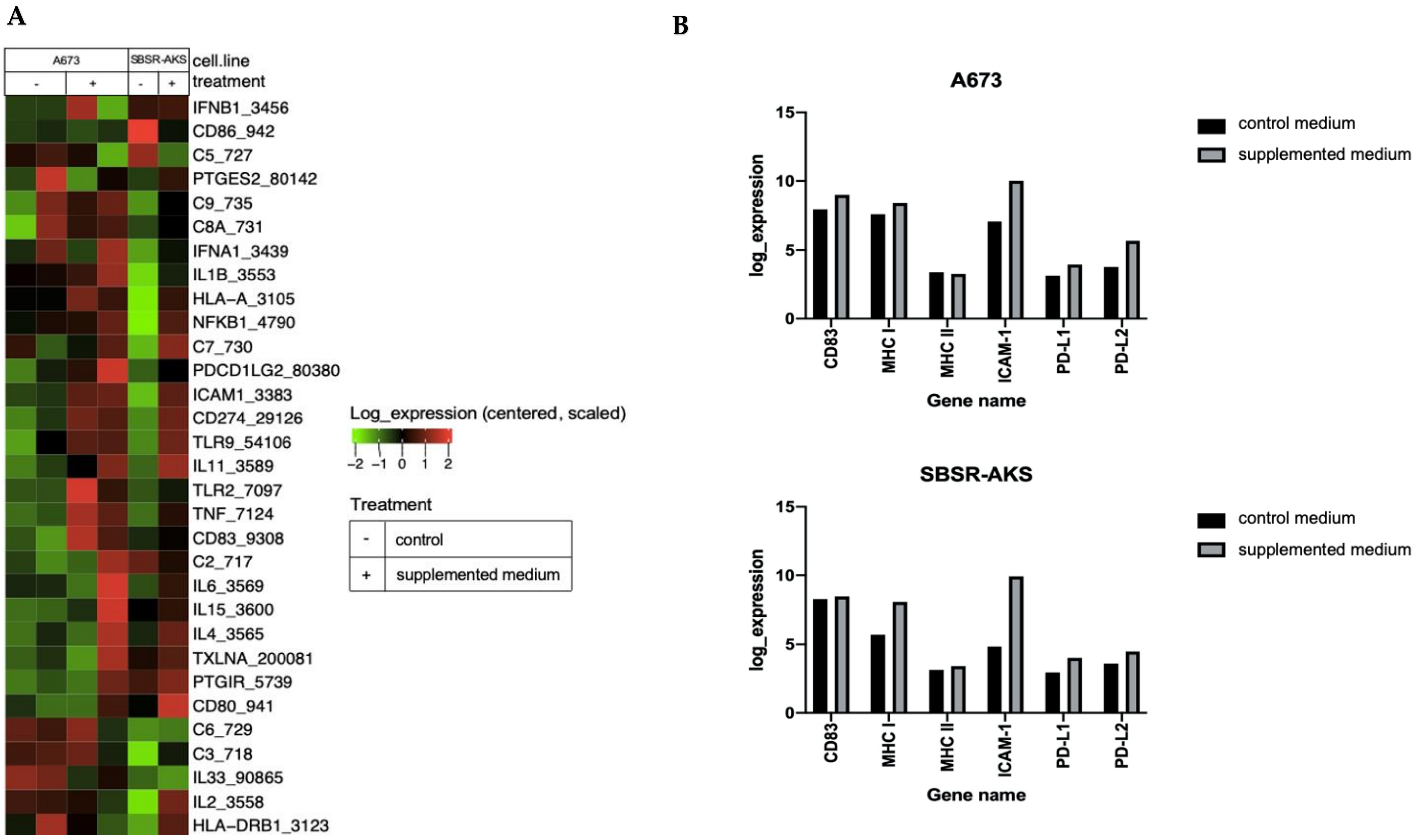

Supplemental Figure S1 - RNA expression. **A)** RNA expression of DC medium supplemented- versus control A673 and SBSR-AKS EwS cell lines; Heatmap analysis particularly reveals a distinct immunogenic RNA expression signature with an increase of CD83, PD-L1 (CD274), TLR2, TLR9 and ICAM-1 in both DC medium supplemented (+) versus control (-) EwS cell lines A673 and SBSR-AKS. **B)** RNA expression of CD83, MHC class I, MHC class II, ICAM-1, PD-L1 and PD-L2 on A673 and SBSR-AKS EwS cell lines. In line with Figure 2A, respective gene expression signature differences are demonstrated between the coontrol (black) A673 and SBSR-AKS controls versus the respective supplemented (grey) EwS cell lines.

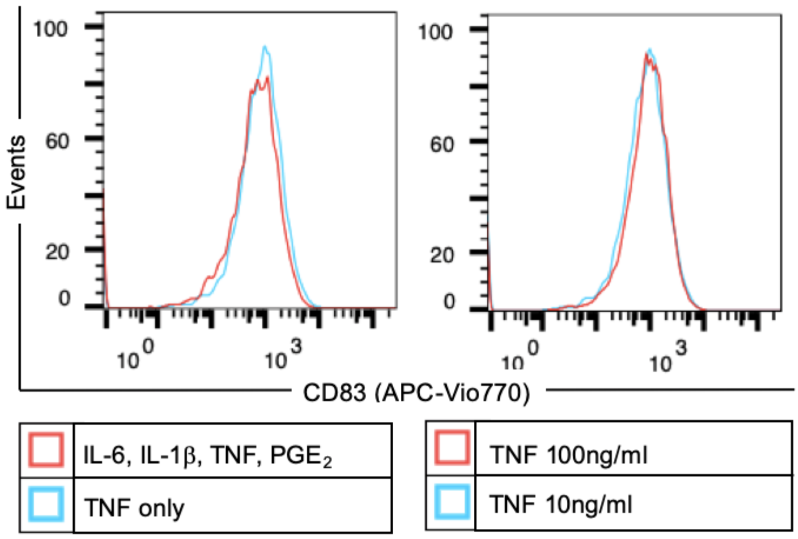

Supplemental Figure S2 – CD83 flow cytometry. Left panel: No Difference in CD83 expression between A673 cells supplemented with only TNF (blue histogram) versus A673 treated with full DC maturation cocktail (red histogram) at day 7 of the DC maturation protocol. Right panel: No Difference in CD83 expres-sion between A673 cells supplemented with standard dose 10µg/ml TNF (blue histogram) versus fivefold 50µg/ml TNF dose (red histogram) at day 6 of the DC maturation protocol (no statistical difference, respec-tively).

A

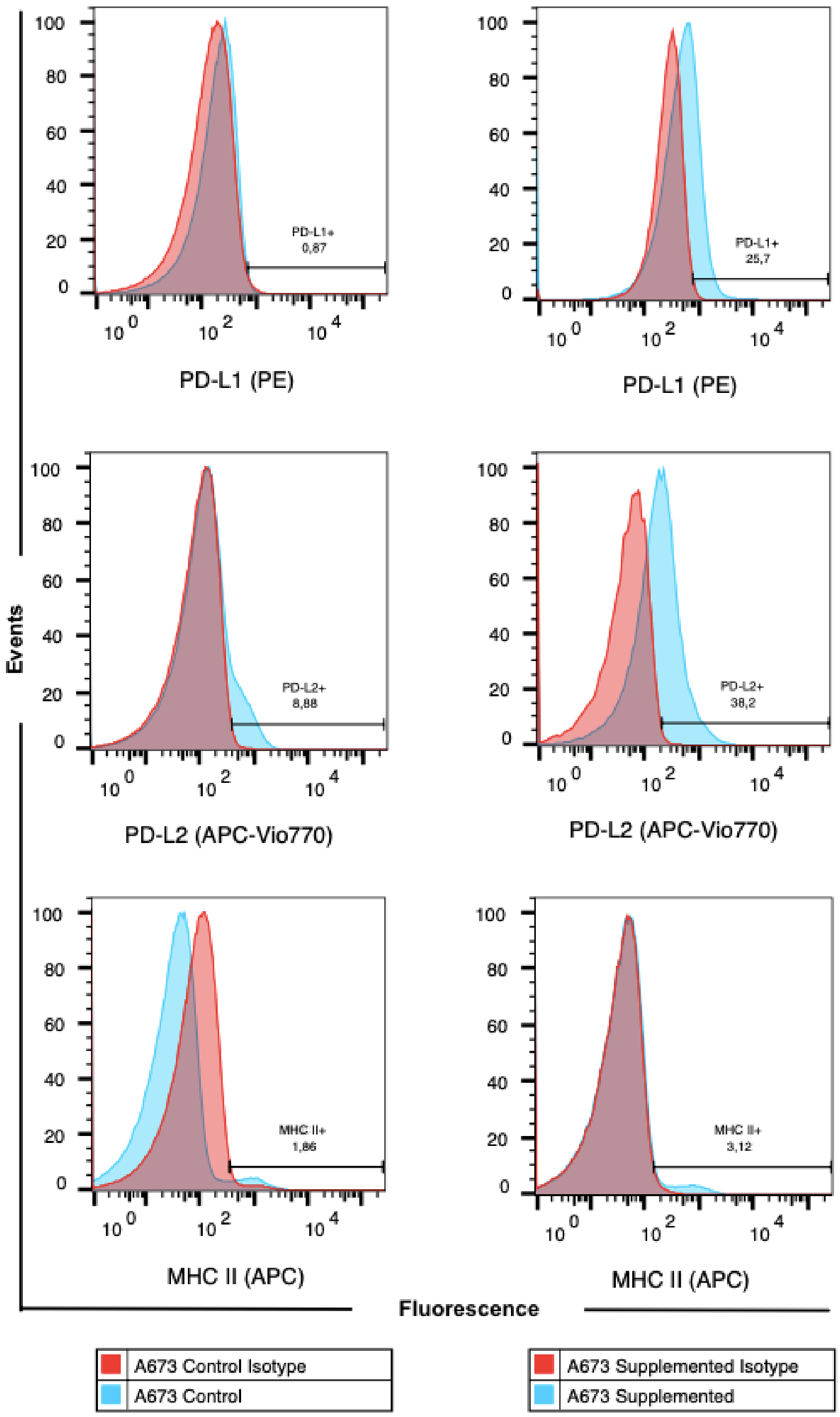
Events
PD-L1+
0,87
PD-L1+
25,7
PD-L1 (PE)
PD-L1 (PE)
PD-L2+
8,88
PD-L2+
38,2
PD-L2 (APC-Vio770)
PD-L2 (APC-Vio770)
MHC II+
1,86
MHC II+
3,12
MHC II (APC)
MHC II (APC)
Fluorescence
A673 Control Isotype
A673 Control
A673 Supplemented Isotype
A673 Supplemented

B

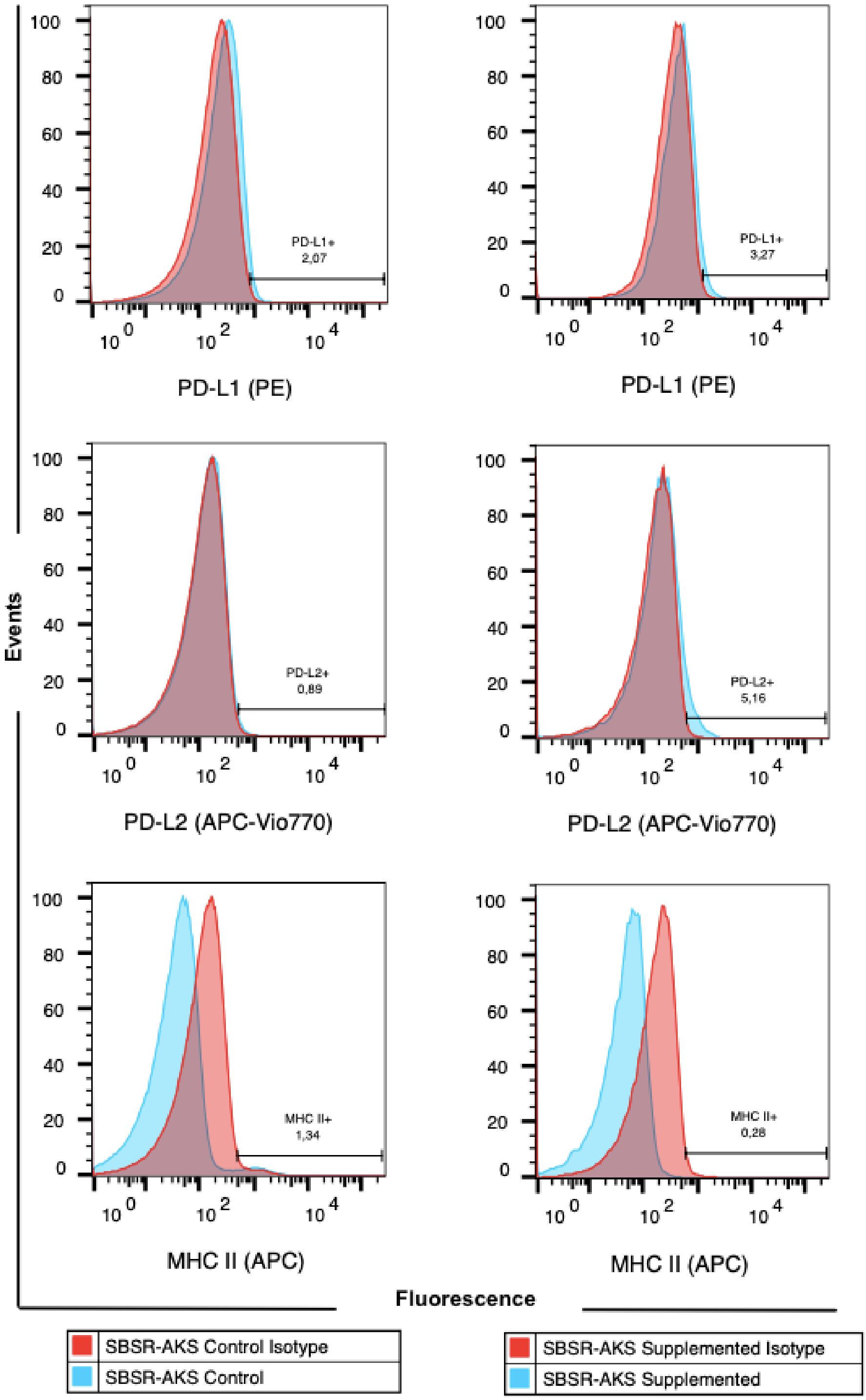

Supplemental Figure S3 – PD-L1, PD-L2 and MHC class II flow cytometry. Figure 2A shows the flow cytometry data of A673 cells stained for PD-L1, PD-L2 and MHC class II comparing supplemented cells versus control cells. All surface expression markers showed a slight increase in the supplemented group, however, the increase is not as marked as for CD83, MHC class I or ICAM-. Figure 2B displays the flow cytometry data of SBSR-AKS cells stained for PD-L1, PD-L2 and MHC class II comparing supplemented cells versus control cells. As demonstrated, there was no increase with any of the surface expression markers.
